# Broad white matter impairment in multiple system atrophy

**DOI:** 10.1002/hbm.25227

**Published:** 2020-10-16

**Authors:** Natalia Del Campo, Owen Phillips, Françoise Ory‐Magne, Christine Brefel‐Courbon, Monique Galitzky, Claire Thalamas, Katherine L. Narr, Shantanu Joshi, Manpreet K. Singh, Patrice Péran, Anne Pavy‐LeTraon, Olivier Rascol

**Affiliations:** ^1^ CHU de Toulouse, Université de Toulouse‐Toulouse 3, INSERM, UMR1214 Toulouse NeuroImaging Centre “TONIC,” Center of Excellence in Neurodegeneration (CoEN), NeuroToul, Centre National de Reference AMS, Centre Expert Parkinson de Toulouse, Centre d'Investigation Clinique CIC1436, Services de Neurologie et de Pharmacologie Clinique, UMR 1048 Institute for Cardiovascular Diseases Toulouse France; ^2^ Division of Child and Adolescent Psychiatry, Department of Psychiatry Stanford University School of Medicine Stanford California USA; ^3^ BrainKey San Francisco California USA; ^4^ Department of Neurology Ahmanson Lovelace Brain Mapping Center, David Geffen School of Medicine at UCLA Los Angeles California USA

**Keywords:** diffusion tensor imaging, MRI, multiple system atrophy

## Abstract

Multiple system atrophy (MSA) is a rare neurodegenerative disorder characterized by the widespread aberrant accumulation of α‐synuclein (α‐syn). MSA differs from other synucleinopathies such as Parkinson's disease (PD) in that α‐syn accumulates primarily in oligodendrocytes, the only source of white matter myelination in the brain. Previous MSA imaging studies have uncovered focal differences in white matter. Here, we sought to build on this work by taking a global perspective on whole brain white matter. In order to do this, in vivo structural imaging and diffusion magnetic resonance imaging were acquired on 26 MSA patients, 26 healthy controls, and 23 PD patients. A refined whole brain approach encompassing the major fiber tracts and the superficial white matter located at the boundary of the cortical mantle was applied. The primary observation was that MSA but not PD patients had whole brain deep and superficial white matter diffusivity abnormalities (*p* < .001). In addition, in MSA patients, these abnormalities were associated with motor (Unified MSA Rating Scale, Part II) and cognitive functions (Mini‐Mental State Examination). The pervasive whole brain abnormalities we observe suggest that there is widespread white matter damage in MSA patients which mirrors the widespread aggregation of α‐syn in oligodendrocytes. Importantly, whole brain white matter abnormalities were associated with clinical symptoms, suggesting that white matter impairment may be more central to MSA than previously thought.

## Graphical abstract

Multiple system atrophy (MSA) differs from other synucleinopathies such as Parkinson's disease (PD) in that α‐synuclein accumulates primarily in oligodendrocytes, the only source of white matter myelination in the brain. In this diffusion tensor imaging study, we observe widespread white matter damage in MSA patients, but not in PD patients, compared to healthy matched controls. Importantly, white matter abnormalities were associated with clinical symptoms, suggesting that white matter impairment may be more central to MSA than previously thought.

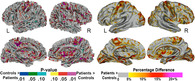

## 1 INTRODUCTION

Multiple system atrophy (MSA) is a rare progressive neurodegenerative disorder characterized clinically by the triad of parkinsonism, cerebellar ataxia, and autonomic failure (Fanciulli & Wenning, 2015). MSA is unique compared to other synucleinopathies in that α‐synuclein (α‐syn) accumulates primarily in oligodendrocytes (Papp, Kahn, & Lantos, 1989), forming glial cytoplasmic inclusions (GCIs) throughout the MSA brain (Inoue et al., 1997; Papp & Lantos, 1994; Trojanowski, Revesz,, & Neuropathology Working Group on MSA, 2007), mostly located in the white matter (Inoue et al., 1997; Papp et al., 1989). Neuropathological and experimental animal evidence suggests that GCIs lead to astrogliosis and microgliosis, demyelination and axonal degeneration, possibly prior to neurodegeneration (Brück, Wenning, Stefanova, & Fellner, 2016; Ettle et al., 2016; Ettle, Schlachetzki, & Winkler, 2016; Grigoletto et al., 2017; Nykjaer, Brudek, Salvesen, & Pakkenberg, 2017; Salvesen et al., 2015a; Salvesen et al., 2015b; Wong, Halliday, & Kim, 2014). Moreover, beyond α‐syn, the relocation of p25α from the myelin sheath to the cytoplasm is probably an early event in the disease process (Song et al., 2007). This relocation leads to significant changes in oligodendroglial morphology and a reduction in smaller diameter myelinated axons. White matter degeneration may thus be a central feature of this disease, in addition to neuronal loss. Diffusion magnetic resonance imaging (MRI) studies have previously reported white matter abnormalities in MSA. For example, regions such as the cerebellar peduncles, pons, and corpus callosum have been found to be particularly vulnerable in MSA, consistent with the clinical manifestation of the disease (see Table S1, Hara et al., 2018; Worker et al., 2014; Zanigni et al., 2017).

We aimed to take a step back and instead of focusing on focal abnormalities we sought to take a global perspective to investigate if there are whole brain white matter abnormalities in MSA. In order to do so, in vivo structural MRI and diffusion tensor imaging (DTI) were performed on MSA patients (*n* = 26), Parkinson's disease (PD) patients (*n* = 23), and controls (*n* = 26). Data were examined using a comprehensive, spatially refined whole brain approach encompassing both the superficial white matter adjacent to the cortical gray matter, and the large deep white matter fiber tracts. We chose to investigate superficial and deep white matter because both have been shown to be vulnerable in neurodegenerative diseases (Phillips, Joshi, Piras, et al., 2016; Phillips, Joshi, Squitieri, et al., 2016b; Rulseh et al., 2016) and have unique properties (see Table S2).

We hypothesized that there would be broad whole brain white matter microstructure abnormalities in MSA patients, but not PD patients, compared to controls. It was also hypothesized that broad whole brain white microstructure abnormalities in MSA patients would be associated with clinical measures.

## 2 MATERIALS AND METHODS

### 2.1 Standard protocol approvals, patient consents, and data availability

The study was approved by the local ethics committee according to French public health legislation (ID RCB 2012‐A01252‐41). All participants provided written informed consent. Assessments were carried out at the Toulouse Clinical Investigation Center and the Toulouse NeuroImaging Center. Study data are currently not available in a public repository.

### 2.2 Subjects

Patients diagnosed with MSA and PD (*n* = 30 per group) were consecutively recruited via the outpatient clinics of the MSA French National Reference Center and the PD Expert Center of Toulouse. Diagnoses were performed by movement disorder specialists (A. P.‐L. T. and O. R.). As described elsewhere (Barbagallo et al., 2016), inclusion criteria for patients were: (a) diagnosis of MSA or PD as per international diagnostic criteria (Gilman et al., 2008); (b) Hoehn and Yahr scores less than 4 on treatment; (c) no history of neurological or psychiatric diseases other than MSA or PD, (d) no evidence of a significant cognitive deficit (Mini‐Mental State Examination [MMSE] score > 24); (e) no prior exposure to deep brain stimulation; (f) no evidence of movement artifacts, vascular brain lesions, or brain tumor. Moreover, 27 sex‐ and age‐matched healthy controls were recruited via the Toulouse Clinical Investigation Center. Overall, 12 subjects (4 MSA, 7 PD, and 1 healthy control) were excluded from analyses due to movement artifacts or severe atrophy as agreed between two of the authors (O. P. and P. P.) after visual inspection before data analysis was performed. Data from the remaining 26 MSA patients, 23 PD patients, and 26 healthy controls were analyzed for the current study. Within the MSA group, 15 were diagnosed with the MSA subtype MSA‐P and 11 with MSA‐C due to exhibiting predominantly parkinsonian versus cerebellar symptoms, respectively.

### 2.3 Motor and cognitive assessments

Motor disabilities in MSA and PD patients were examined using the Motor Examination scores of the Unified MSA Rating Scale, Part II (UMSARS‐II) and the Unified Parkinson's Disease Rating Scale, Part III (UPDRS‐III), the reference scales to assess motor function in MSA and PD, respectively (Fahn, Elton,, & Members of the UPDRS Development Committee, 1987; Wenning et al., 2004). Cognition was assessed in all subjects using the MMSE (Folstein, Folstein, & McHugh, 1975).

### 2.4 Image acquisition

Images were acquired on a 3‐Tesla MRI scanner (Intera Achieva, Philips, Best, the Netherlands). A three‐dimensional T1‐weighted sequence (in‐place resolution 1 × 1 mm; slice thickness 1 mm; 160 contiguous slices) was obtained for each subject. Diffusion‐weighted volume measurements were also acquired using a spin‐echo echo‐planar imaging (TE/TR555/11,031 ms; bandwidth = 3,026.4 Hz/voxel; matrix 5,108 × 106; 95 slices; voxel size 1.8 × 1.8 × 1.8 mm) with 32 isotropically distributed orientations for the diffusion‐sensitizing gradients at a *b* value of 1,000 s/mm^2^ and *b* = 0 images.

### 2.5 Image processing

Two diffusion images methods were chosen to examine the white matter: (a) superficial white matter mapping (Phillips et al., 2011, 2013, 2014, 2018; Phillips, Joshi, Piras, et al., 2016) in order to examine the boundary between the cortical gray matter and deep white matter and (b) tract‐based spatial statistics (TBSS) in order to examine the deep white matter (Smith et al., 2006):

#### 2.5.1 Superficial white matter

In brief, T1‐weighted images were processed using BrainSuite's cortical surface extraction pipeline (http://brainsuite.org/processing/surfaceextraction/, v16), which produces surface models of the cerebral cortex from T1 MRI (Shattuck & Leahy, 2002). Next, the surfaces for each subject were registered to a reference atlas surface using BrainSuite's surface/volume registration software (SVReg; http://brainsuite.org/processing/svreg/) (Joshi & Shattuck, 2012; Joshi, Shattuck, Thompson, & Leahy, 2004, 2007). Outputs from SVReg were inspected to ensure proper segmentation and surface/volume registration. As an additional quality control for the diffusion data, we used DTIPrep (https://www.nitrc.org/projects/dtiprep/), a program that is designed to address data quality problems that affect diffusion MRI, including movement artifacts. All subjects included in this study passed quality control using the standard DTIPrep threshold. The diffusion‐weighted images were processed with the BrainSuite Diffusion Pipeline (BDP; Bhushan et al., 2015; http://brainsuite.org/processing/diffusion/). BDP was used to correct for geometric distortions in diffusion images (registration‐based distortion correction) and to co‐registers diffusion and anatomical images. BDP registrations were individually inspected (by O. P.) to ensure a satisfactory registration.

Region of interest labels from the SVReg output (specifically, the caudate, thalamus, putamen and cerebellum) were then applied to the mean diffusivity image. Finally, to allow cross‐subject sampling of anatomically comparable superficial white matter mean diffusivity, diffusivity was sampled along each vertex of the white matter surface that had been mapped to the atlas reference via SVReg. In order to extract the whole brain superficial white matter mean diffusivity for each subject, the diffusivity value at each vertex was then extracted and the mean value from across these vertices was calculated.

#### 2.5.2 Deep white matter

FMRIB Software Library (FSL‐TBSS) was run (Smith et al., 2006). In short, data were subjected to eddy current and motion correction, followed by brain extraction. Dtifit was used to generate mean diffusivity, fractional anisotropy (FA), radial diffusivity, and axial diffusivity. A study‐specific mean FA skeleton representing the centers of all white matter tracts was generated and used to transform all subjects to MNI152 space. The white matter skeleton was thresholded at 0.2. The mean FA skeleton was then binarized and applied to each subject's mean diffusivity image, which allowed the extraction of the whole brain mean diffusivity value.

### 2.6 Selection of diffusion parameter

The parameter chosen to be the focus of this study was mean diffusivity, which is the mean of the three eigenvalues and corresponds to the molecular diffusion rate (lower values mean low diffusivity) (Soares, Marques, Alves, & Sousa, 2013). Mean diffusivity was chosen rather than other diffusion metrics such as FA and axial/radial diffusivity for a number of reasons. One, to reduce the number of tests. Two, mean diffusivity has been demonstrated to be a sensitive biomarker in both the deep white matter and in the superficial white matter (Clark et al., 2011; Narr et al., 2009; Phillips, Joshi, Squitieri, et al., 2016b). Three, the difference between axial and radial diffusivity values is considerably smaller in the superficial white matter than in the deep white matter (Phillips et al., 2013; Phillips, Joshi, Piras, et al., 2016; Phillips, Joshi, Squitieri, et al., 2016b). This reduces the usefulness of individually investigating these measures and FA.

Mean diffusivity is on average lower in the deep white which reflects the restricted diffusion and generally greater coherence of white matter connections in a particular voxel while the mean diffusivity is on average higher in the superficial white matter which reflects the greater diffusion and generally increased tissue complexity within a particular voxel. The mean diffusivity within the superficial white matter is higher because it contains cortico‐cortical short‐range connections, long‐range axonal projections, as well as a high proportion of “interstitial neurons.” However, volumetrically, the superficial white matter is largely composed of myelin and oligodendrocytes (Phillips et al., 2011; Reveley et al., 2015; Suárez‐Solá et al., 2009).

### 2.7 Statistical analysis

#### 2.7.1 Whole brain white matter group differences

Analyses were performed using SPSS Statistics 22. Whole brain mean diffusivity for the deep and superficial white matter was used to test for differences between groups (MSA vs. controls, PD vs. controls) using SPSS's general linear model (GLM) with sex and age as covariates.

#### 2.7.2 Correlations between whole brain diffusivity and clinical measures

To investigate whether superficial and deep white matter changes in MSA patients were related to UMSARS‐II and MMSE, we ran a partial correlation analysis within SPSS between these measures and whole brain superficial and deep white matter mean diffusivity with sex and age as covariates. Based on robust a priori hypotheses about directionality of these correlations (given the prerequisite of previously established group differences), correlations were performed one tailed. Levels of significance were set at alpha *=* 0.05.

#### 2.7.3 Exploratory vertex‐ and voxel‐based analysis

For increased spatial resolution, we applied an exploratory vertex (GLM (http://brainsuite.org/bss/) (Joshi et al., 2014). A vertex‐based analysis was used for the superficial white matter because the analysis uses 3D surface meshes. For the deep white matter, a voxel (TBSS Randomize) analysis based on the image data was done between MSA patients and controls with sex and age included as covariates.

## 3 RESULTS

### 3.1 Demographic and clinical characteristics

The study included 26 patients diagnosed with MSA, 23 patients diagnosed with PD, and 26 controls. Demographic and clinical characteristics are shown in Table 1. There were no significant differences in terms of age or gender between MSA patients, PD patients, and controls (all *p* > .05), nor between MSA‐P and MSA‐C patients. Years since disease onsets were also similar between diagnostic groups.

**TABLE 1:** Demographic and clinical characteristics of the three main groups (controls, PD and MSA patients)

Group	Controls	PD	MSA
N	26	23	26
Female/male ratio	15/11	11/12	15/11
Age (years)	66 ± 5	64 ± 7	64 ± 8
Years since disease onset (years)		7 ± 4	6 ± 2
UMSARS‐II			30 ± 8
UPDRS‐III		19 ± 10	
MMSE	29 ± 1	29 ± 1	28 ± 2

*Note:* Data are mean ± *SD*.

Abbreviations: MMSE, Mini‐Mental State Examination; MSA, multiple system atrophy; MSA‐C, MSA subtype associated predominantly with cerebellar symptoms; MSA‐P, MSA subtype associated predominantly with parkinsonian symptoms; PD, Parkinson's disease; UMSARS‐II, motor score of the Unified MSA Rating Scale, Part II; UPDRS‐III, motor score of the Unified Parkinson's Disease Rating Scale, Part III.

### 3.2 Whole brain white matter differences in MSA versus controls

**FIGURE 1:**
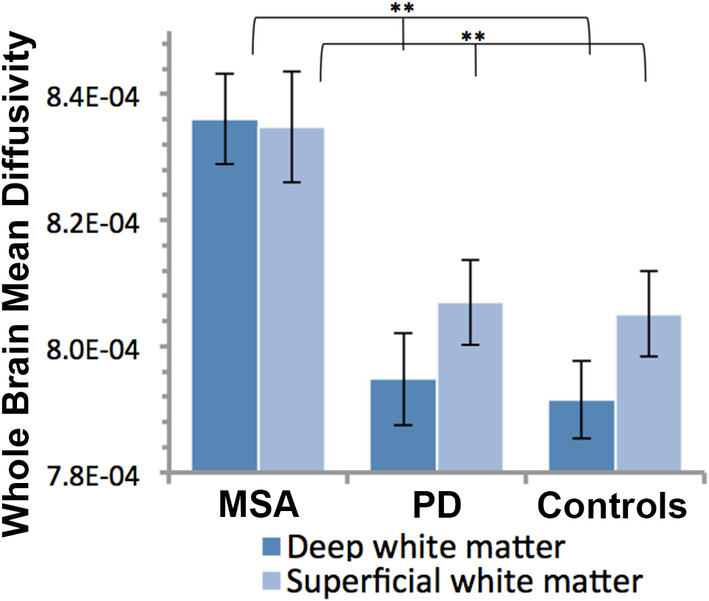
Mean diffusivity in deep and superficial white matter over the whole brain in MSA patients, PD patients and controls. **p < .01. Error bars are *SEM*. MSA, multiple system atrophy; PD, Parkinson's disease

As shown in Figure 1, MSA had increased global mean diffusivity compared to controls in both superficial matter and deep white matter (*F*[3,48] = 16.72, *p* < .001 and *F*[3,48] = 33.01, *p* < .001, respectively).

**FIGURE 2:**
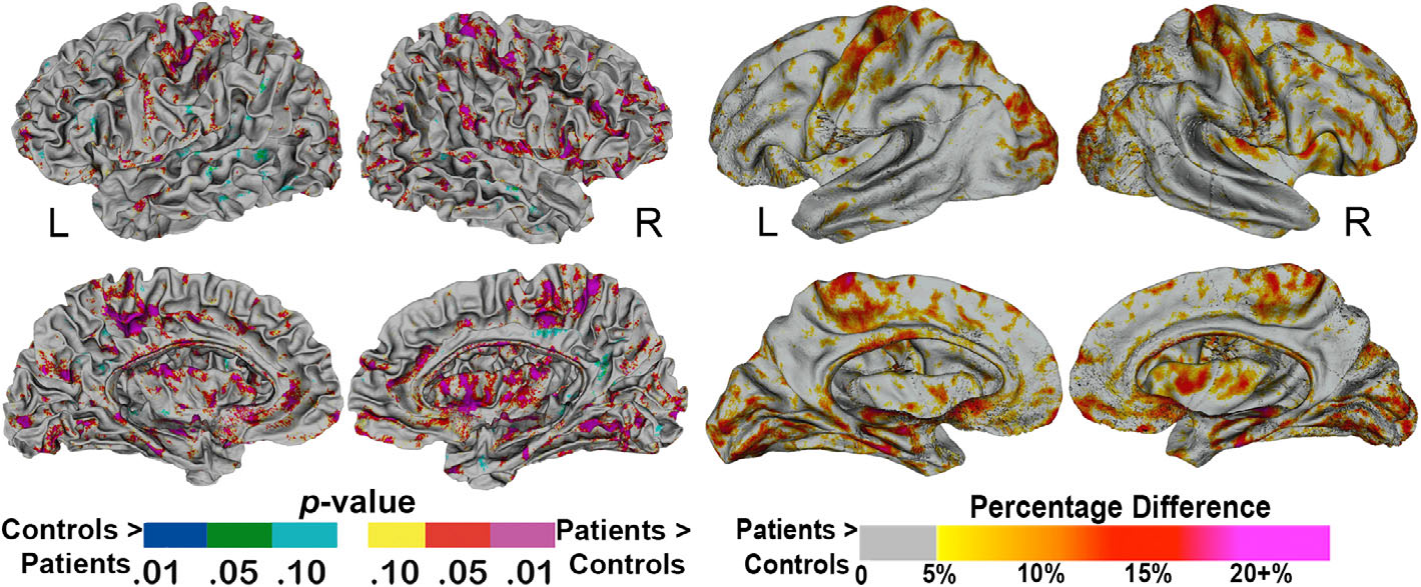
Vertex‐based differences in the superficial white matter between controls and MSA patients. Color maps represent level of significance (*p* values) on the left, and percentage difference on the right. Results are uncorrected for multiple comparisons. MSA, multiple system atrophy

**FIGURE 3:**
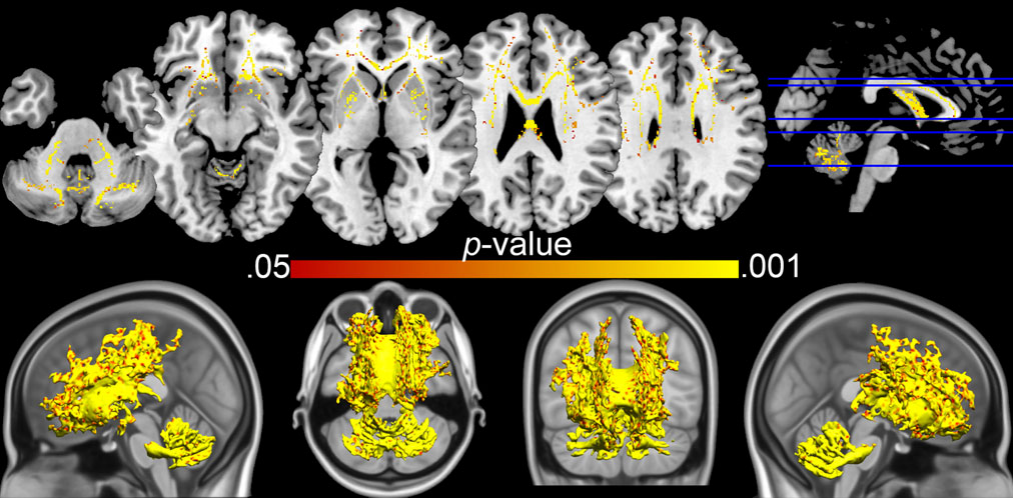
TBSS results—MSA versus healthy controls. Clusters shown are all *p* < .05 (Threshold‐free cluster enhancement corrected). MSA, multiple system atrophy; TBSS, tract‐based spatial statistics

In order to examine the specific location of white matter changes in the brain exploratory vertex and voxel‐wise analyses were conducted. They revealed that mean diffusivity levels were increased in the MSA group throughout the brain, as can be observed in Figure 2 for the superficial white matter and Figure 3 for the deep white matter. The widely distributed TBSS clusters shown in Figure 3 survived Threshold‐Free Cluster Enhancement and correction for multiple comparisons (*p* < .05). For the superficial white matter effects did not survive vertex false discovery rate correction. On the basis of the established group differences in global superficial white matter, uncorrected results are shown in Figure 2.

### 3.3 White matter differences in PD versus controls

In contrast to the marked increase in whole brain white matter mean diffusivity observed in MSA patients compared to controls, this difference was not observed for PD patients (Figure 1). Vertex‐ and voxel‐based analyses exploring further potential regional effects confirmed this finding (results not shown).

### 3.4 Relationships between white matter mean diffusivity and clinical variables in MSA

**FIGURE 4:**
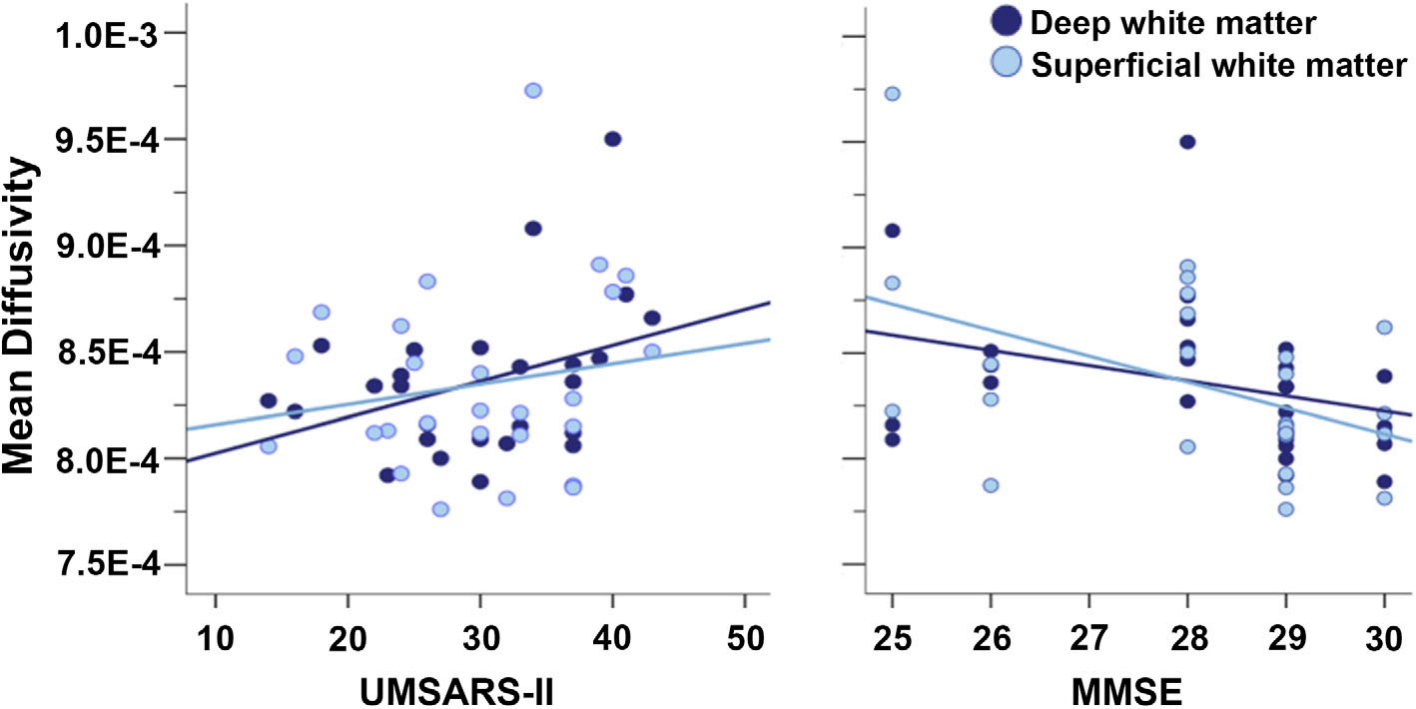
Whole brain mean diffusivity correlation with clinical measures within MSA patients. MMSE, Mini‐Mental State Examination; MSA, multiple system atrophy; UMSARS, Unified Multiple System Atrophy Rating Scale; WM, white matter

Correlation analyses explored whether the increased whole brain white matter mean diffusivity observed in MSA patients was associated with their clinical profile. As shown in Figure 4, whole brain white matter mean diffusivity was significantly correlated with both motor and cognitive functions. More specifically, scores on the UMSARS motor subscale were positively correlated with whole brain deep and superficial white matter mean diffusivity (*r* = .53, *p* < .001 and *r* = .39, *p* = .03, respectively). The MMSE scores were negatively correlated with whole brain superficial white matter mean diffusivity (*r* = −.46, *p* = .01), while a trend correlation, also negative, was observed with deep white matter mean diffusivity (*r* = −.30, .079). The opposite directionality of the correlations with motor and cognitive function was expected, as dysfunction is indexed by higher scores on the UMSARS and lower scores on the MMSE.

## 4 DISCUSSION

The main finding was that MSA but not PD patients had widespread deep and superficial white matter microstructure abnormalities compared to controls. In MSA patients, whole brain deep and superficial white matter diffusion was associated with clinical measures, including motor dysfunction, suggesting that pervasive white matter damage underlies some of the symptomatic manifestations of MSA.

### 4.1 Widespread white matter abnormalities in MSA

Our results suggest that there are pervasive white matter abnormalities in MSA. First, large whole brain differences in mean diffusivity were observed between MSA patients and controls in both deep matter and superficial white matter (Figure 1). These measures were calculated using two different methodological approaches and these dual observations both confirmed the widespread distribution of the white matter abnormalities over the brain. At the vertex and voxel levels, abnormalities were particularly prominent in white matter in the primary motor cortex, cerebellar peduncles, cerebellum, and the frontal lobe, which are among the brain regions known to be GCI rich in MSA (Papp et al., 1989). However, abnormalities were not limited to those regions, just as neuropathological studies have reported GCIs beyond those regions as well (Papp et al., 1989). Second, whole brain diffusivity in MSA was correlated with two independent, functional measures of motor and cognitive function, and this was true for diffusion measures of deep and superficial white matter alike. These correlations corroborate the broad white matter abnormality in MSA, and imply that this abnormality is central to the clinical manifestation of the disease. Broad superficial white matter abnormalities have also been shown to be associated with cognitive function in Alzheimer's disease (Phillips et al., 2013) Huntington's disease (Phillips, Joshi, Squitieri, et al., 2016a) and anti‐ N‐methyl‐D‐aspartate receptor encephalitis (Phillips et al., 2018), which indicates that it may be a sensitive marker of broad nonspecific cognitive dysfunction.

The above group differences and correlations converge to suggest that abnormal mean diffusivity in the superficial and deep white matter are equally pathognomic to MSA. We were interested in both types of white matter tissue for the sake of providing a spatially comprehensive framework, but also because of their distinct cellular specificities; for example, myelination of superficial white matter occurs later than that of deep white matter, which makes superficial white matter particularly prone to lesions. Increasing literature suggests that superficial white matter is an important biomarker showing abnormalities in various neuropathologies, ranging from Alzheimer's disease, through Huntington's disease to schizophrenia or autism (for a review see Guevara, Guevara, Román, & Mangin, 2020). However, compared to deep white matter, superficial white matter has only relatively recently started to be documented in clinical studies, and the differential involvement of superficial versus deep white matter in the pathogenesis of brain disorders remains largely unexplored. Despite the cellular specificities, superficial matter and deep white matter are largely governed by the same mechanisms and are thus likely interdependent at some levels. Further studies are required to elucidate the interdependencies and distinct roles of superficial and deep white matter across neuropathologies but also in the healthy brain.

### 4.2 In vivo MRI in MSA: Interpretation of results and prior literature

The results provide support for our primary hypothesis that MSA would be characterized by widespread white matter abnormalities. in vivo MRI is a limited modality at uncovering the precise nature of individual cellular processes compared to intensive lab benchwork, such as immunochemistry, cell‐based or postmortem cell staining which has been used to identify the distribution of GCIs in the brain (Papp et al., 1989; Papp & Lantos, 1994). However, DTI is a practical methodology to achieve a broad in vivo overview of the white matter. This quality of MRI makes it ideally suited to investigate our hypothesis since white matter is made up primarily of oligodendrocytes, and white matter microstructure can be assessed based on directionality of the overall movement of water molecules as measured by diffusion MRI. Moreover, ex vivo DTI studies coupled with histological assessments have described some of the processes which may underlie changes in DTI parameters, including demyelination (Yano et al., 2017).

White matter microstructure abnormalities have been previously documented in MSA using DTI (Table S1). Most of these prior investigations focused their investigations on predefined brain regions, although more recent work has also reported widespread abnormalities using TBSS (Hara et al., 2018; Rulseh et al., 2016). This research has been helpful in identifying particularly vulnerable regions such as the cerebellar peduncles, pons, and corpus callosum, consistent with the clinical manifestation of MSA (Hara et al., 2018; Worker et al., 2014; Zanigni et al., 2017). Though the previous focal approaches are clearly informative and have like our own study demonstrated abnormalities in white matter in MSA (Table S1), the novelty of our findings lies in the whole brain topography of these white matter abnormalities, which appear more pervasive than previously thought. It is possible that white matter abnormalities in MSA are initially highly localized in vulnerable brain regions and then expand as the disease evolves. An alternative explanation is that the white matter abnormalities are persistent from the beginning, and that these become increasingly severe as the disease evolves. With our current design, we are not able to determine the origins of this pervasiveness.

### 4.3 Specificity of white matter damage: MSA versus PD

Our second hypothesis was that widespread white matter abnormalities would be specific to MSA. The underlying rationale was that MSA is unique in that the accumulation of α‐syn occurs in oligodendrocytes, whereas in PD, α‐syn accumulates in neurons. As expected, no global white matter damage was observed in PD compared to controls. These findings are in line with recent well‐powered DTI studies suggesting that the white matter is largely intact in PD, although focal abnormalities, particularly in the substantia nigra, have been consistently reported (Atkinson‐Clement, Pinto, Eusebio, & Coulon, 2017; Burciu et al., 2017).

### 4.4 Further evidence from the literature supporting widespread white matter damage in MSA

Although the current design does not allow us to investigate the underpinnings of the observed white matter abnormalities, we can draw from the existing neuropathological and experimental evidence in MSA to formulate working hypotheses for future research. A likely neuropathological process contributing to the pervasive white matter abnormality is the hallmark signature of MSA: the accumulation of α‐syn in oligodendrocytes in the form of GCIs. Postmortem and experimental evidence suggests that GCIs lead to astrogliosis and microgliosis, demyelination, and axonal degeneration (Bassil et al., 2017; Ettle, Schlachetzki, & Winkler, 2016; Nykjaer et al., 2017; Yazawa et al., 2005), which would be expected to compromise the integrity of white matter microstructure. Changes in oligodendroglial morphology and axonal myelin have been observed even before GCIs are developed (Song et al., 2007). Moreover, the widespread nature of our diffusion results largely mirror the distribution of GCIs found in *postmortem* studies, where GCIs have been found in the deep white matter fibers of the putamen, the internal capsule, external capsule, corpus callosum, anterior commissure, corticopontine tract, pyramidal tract, cerebellar white matter, and the superficial white matter (neocortex cortical layers five and six) and the frontal lobe (primary motor, premotor areas, and supplementary motor cortical area) (Papp et al., 1989; Papp & Lantos, 1994).

Previous studies have established that insoluble α‐syn aggregates eventually lead to cell death (Bassil et al., 2017; Yazawa et al., 2005). For example, transgenic mice that overexpress wild‐type human α‐syn specifically in oligodendrocytes develop GCI‐like α‐syn deposits and exhibit loss of oligodendrocytes and neurons (Yazawa et al., 2005). However, it has been suggested that there is widespread oligodendrocyte dysfunction early or even initially during MSA pathogenesis, prior to cell loss (Ettle, Schlachetzki, & Winkler, 2016). Recent neuropathological evidence collected in MSA patients comparable to our own in terms of age and disease duration shows largely preserved oligodendrocyte numbers in the presence of significant microgliosis, astrocytosis and neuronal loss, both sub‐cortically (Salvesen et al., 2015a) and in the neocortex (Salvesen et al., 2015b). The present investigation examined the white matter supporting the deep subcortical and cortical gray matter where these widespread neuronal, astrocytes and microglia abnormalities were described.

There are other neurobiological events co‐occurring alongside the putative oligodendrocyte dysfunction which may further contribute to white matter abnormalities in MSA patients. First, astrocytic and microglial activation has been previously reported in MSA (Vieira, Radford, Chung, Guillemin, & Pountney, 2015). For example, elevated tumor necrosis factor alpha and interleukin 1 beta have been observed in prefrontal cortex tissue samples from patients with MSA, pointing to disease‐related neuroinflammation (Salvesen et al., 2015a; Salvesen, Winge, et al., 2015). Second, loss of interstitial neurons, a further pathological characteristic of MSA (Nykjaer et al., 2017), may also have an impact on white matter microstructure. Although the role of interstitial neurons in MSA is unknown, they are located in the white matter, most abundantly in the superficial layers (Suárez‐Solá et al., 2009).

The combined effect of the above interlinked events, including widespread oligodendrocyte dysfunction, oligodendrocyte cell death, neuroinflammatory responses, and loss of interstitial neurons may contribute to the widespread diffusion changes we observe in the present study. Although speculative at this point, it is not unreasonable to think that oligodendrocyte dysfunction is likely the most prominent underpinning of all, since (a) GCIs containing aggregated α‐syn are pathognomonic of MSA and (b) previous neuropathological studies found largely preserved oligodendrocyte cell counts in MSA patients comparable to our own in terms of age and disease stage.

### 4.5 Limitations

The first limitation is the relatively reduced sample size, which results from the challenge of recruiting patients with this rare disease. Nevertheless, this is the largest in vivo white matter study in MSA patients to date. Although MRI does not allow identifying the neural substrates of observed abnormalities, we formulate hypotheses grounded in the neuropathological and experimental literature for future research to (a) definitely confirm that white matter changes are driven by α‐syn accumulation in oligodendrocytes, for example, obtaining histopathological evidence of scanned patients, and (b) examine potential relationships with concomitant neurodegeneration. An important limitation of performing MRI studies on patients with movement disorders is the inherent problem that patients can often move in the scanners. Therefore, it is possible that artifacts due to head movement in patients could impact the results in this study. DTI data are particularly sensitive to movement. For example, in a study on autism (Koldewyn et al., 2014) diffusion results were greatly reduced when subjects were matched for movement. To minimize the impact of motion artifacts we performed extra quality control steps. We used MMSE scores to explore potential associations between white matter integrity and cognitive function. Although it is a well‐established measure of global cognition commonly used in other neurodegenerative diseases, the MMSE is seldom used in parkinsonism and thus should be interpreted with caution. This examination was chosen because MMSE scores have been previously shown to correlate with whole brain superficial white matter diffusivity in other neurodegenerative diseases, strengthening our findings (Phillips et al., 2013). A further limitation is that with our current design we cannot determine to what extent the pervasive abnormalities we observe in MSA patients are focal to begin with and progressively expand throughout the brain. Future work needs to address these questions using early and late stage MSA patients, and reliable markers of disease progression.

## 5 CONCLUSIONS

We find strong evidence for pervasive white matter abnormalities in MSA, but not PD, suggesting that white matter dysfunction may be more central to MSA than previously thought. The fact that white matter abnormalities were specific to MSA raises the possibility that these are related to the presence of GCIs, which are pathognomic to the disease. The link between white matter integrity in MSA and oligodendrocyte (dys)function has been hitherto largely neglected in clinical research. We discuss some of the mechanisms which likely underlie the observed white matter abnormalities in MSA in the light of existing neuropathological and experimental evidence. Further studies are needed to definitely confirm that white matter changes are driven by GCIs and their downstream effects and examine potential relationships with concomitant neurodegeneration.

## CONFLICT OF INTERESTS

Owen Phillips receives support from BrainKey. Manpreet K. Singh receives research support from Stanford's Child Health Research Institute, National Institute of Mental Health, Office of Research on Women's Health, National Institute of Aging, Neuronetics, Johnson and Johnson, and the Brain and Behavior Foundation. She has been on the advisory board for Sunovion. Olivier Rascol receives funding from Agence Nationale de la Recherche (ANR), CHU de Toulouse, France‐Parkinson, INSERM‐DHOS Recherche Clinique Translationnelle, MJFox Foundation, Programme Hospitalier de Recherche Clinique and European Commission (FP7, H2020). He has also received funding from the International Movement Disorders Society to participate in symposia and contribute to reviews. He is on the advisory board of AbbVie, Adamas, Acorda, Addex, AlzProtect, Apopharma, Astrazeneca, Bial, Biogen, Britannia, Clevexel, INC Reasearch, Lundbeck, Lupin, Merck, MundiPharma, Neuratris, Neuroderm, Novartis, Oxford Biomedica, Parexel, Pfizer, Prexton Therapeutics, Quintiles, Sanofi, Servier, Sunovion, Takeda, Teva, UCB, XenoPort and Zambon. Natalia Del Campo, Françoise Ory‐Magne, Christine Brefel‐Courbon, Monique Galitzky, Claire Thalamas, Katherine L. Narr, Shantanu Joshi, Patrice Péran, and Anne Pavy‐LeTraon have no conflicts of interest to report.

## Supporting information


**Table S1** Diffusion tensor imaging studies on MSA.
**Table S2**. Differences between superficial and deep white matter.

## Data Availability

Study data are currently not available in a public repository.
