# Supplementary material for: Broad white matter impairment in multiple system atrophy
Source: Hum Brain Mapp. 2020 Oct 16;42(2):357–66. doi: 10.1002/hbm.25227 (PMC7776008; doi:10.1002/hbm.25227)
Supplement: Supplementary file 1 — Table S1 Diffusion tensor imaging studies on MSA. Table S2. Differences between superficial and deep white matter. [file HBM-42-357-s001.docx]

## Supporting information

#### **Table S1: Diffusion tensor imaging studies on MSA.**

#### Only studies including a minimum of 10 MSA patients and controls were included.

| Authors | DTI method | Groups (N) | Age of MSA patients years (SD) | Key results (MSA vs. controls) |
| --- | --- | --- | --- | --- |
| Zanigni 2017 ^1^ | TBSS, tractography | MSA-C (9) and MSA-P (9), PSP (25), PD (47), controls (27) | MSA-C: 59 (49-69) ^a^, MSA-P: 64 (41-72) ^a^ | Decreased FA and increased mean diffusivity in MSA-C in middle cerebellar peduncles. |
| Chiang 2017 ^2^ | TBSS | MSA-P (20), PD (18), controls (24) | 61 (5) | Decreased FA in the middle cerebellar peduncle, pontine crossing tract and corticospinal tract bilaterally. |
| Du 2017 ^3^ | ROI analysis (striatal, midbrain, limbic and cerebellar ROIs) | MSA-P (16), PD (35), PSP(19) Controls (36) | 68 (8) | Increased mean diffusivity and decreased FA across most ROIS examined. |
| Wang 2017 ^4^ | Tractography | MSA-C (20), controls (30) | 54 (N/A) | Reduced structural connectivity in cortical regions such as the insula, anterior cingulate gyrus, supplementary motor area, cuneus, precuneus, and cerebellum. |
| Rulseh 2016 ^5^ | TBSS | MSA (20), controls (20) | 61 (7) | Widespread increased mean diffusivity, decreased FA, increased radial diffusivity and increased axial diffusivity. |
| Yang H 2015 ^6^ | ROI analysis (cerebellar peduncles, posterior limbs of the internal capsule and pontine crossing tract) | MSA-C (30), controls (30) | 55 (9) | Reduced FA and increased MD across all ROIs. |
| Worker 2014 ^7^ | TBSS | MSA (17), PD (14), PSP (16), controls (18) | 62 (7) | Increased mean diffusivity in the corticospinal tract, middle and inferior cerebellar peduncles and medial lemniscus. |
| Prodoehl 2013 ^8^ | ROI analysis (striatal ROIs, midbrain, red nucleus, cerebellar peduncles and dentate nucleus) | MSA-P (14), PD (15), PSP (12), essential tremor (14) and controls (17) | 64 (9) | Comparison MSA vs. controls not reported. |
| Surova 2013 ^9^ | Tractography | MSA-P (12), PD (10), PSP (16), controls (16) | 63 (N/A) | Increased FA and radial diffusivity in the cingulum and the corticospinal tract. |
| Tsukamoto 2012 ^10^ | ROI analysis (midbrain, pons, putamen, globus pallidus, caudate nucleus, thalamus, cerebellar ROIs) | MSA (25), PSP (20), PD (17) and controls (18) | 65 (8) | Increased apparent diffusion coefficient in the pons, middle cerebellar peduncle, cerebellar white matter, cerebellar dentate nucleus, posterior putamen and midbrain. |
| Focke 2011 ^11^ | ROI analysis in basal ganglia | MSA-P (10), PD (12), PSP (9), controls (13) | 63 (9) | No differences in MD or FA. |
| Tha 2010 ^12^ | Voxelwise analysis of FA and mean diffusivity | MSA-C (16), controls (16) | 60 (5) | Widespread increased mean diffusivity and decreased FA. Significant correlations between diffusion parameters in multiple areas and activities of daily living, ataxia, severity of orthostatic hypotension and disease severity. |
| Pellecchia 2009 ^13^ | ROI analysis (putamen, cerebellar white matter, pons, middle cerebellar peduncle, caudate, putamen | MSA-P (9), MSA-C (12), controls (11) | MSA-P: 69 (7), MSA-C: 58 (5) | Reduced regional trace value in MSA-P patients in the putamen and in MSA-C patients in the cerebellar white matter, pons and middle cerebellar peduncle. |
| Ito 2008 ^14^ | Tractography | MSA (20), ALS (28), controls (17) | 61 (9) | Decreased FA in internal capsule, corona radiate and whole pyramidal tract. |
| Nicoletti 2008 ^15^ | ROI analysis (superior cerebellar peduncle) | MSA-P (15), PD (15), PSP (28), controls (16) | 65 (5) | N/A (see Nicoletti 2006) |
| Seppi 2006 ^16^ | ROI analysis(anterior and posterior putamen) | MSA-P (15), PD (20), controls (11) | 64 (6) | Increased apparent trace of the diffusion tensor in the putamen, particularly in the posterior putamen. |
| Blain 2006 ^17^ | ROI analysis (cerebellar peduncles and pons) | MSA (17), PSP (17), PD (12), controls (12) | 64 (8) | Increased MD and FA across ROIs. |
| Nicoletti 2006 ^18^ | ROI analysis (basal ganglia, thalamus, white matter, pons and middle cerebellar peduncles) | MSA-P (16), PSP (16), PD(16), controls (15) | 65 (5) | Increased regional apparent diffusion coefficient in the middle cerebellar peduncles. |
| Shiga 2005 ^19^ | ROI analysis (cerebellar peduncles, basis pontis, internal capsule and corpus callosum) | MSA (11), controls (10) | 64 (5) | Decreased FA in the middle cerebellar peduncle, basis pontis and internal capsule. |

^a^ Age expressed in median (range); ROI = region of interest, FA = fractional anisotropy, mean diffusivity = mean diffusivity, TBSS = tract-based spatial statistics, PSP = Progressive Supranuclear Palsy, Where MSA sub-types are not specified, both MSA-P and PSA-C patients were included.

### Table S2. Differences between superficial and deep white matter.

| **Superficial White Matter** | **Deep White Matter** |
| --- | --- |
| Short fibers | Long fibers |
| Small diameter fibers | Larger diameter fibers |
| Intracortical connections | Intrahemisheric and interhemisheric connections |
| Late myelinating | Early myelinating compared to SWM |
| Ogliodendrocytes wrap many axons segments | Ogliodendrocytes wrap few axon segments |
| Less myelin wraps around the axon | More myelin wraps around the axon |
| Complex arrangement | Less complex arrangement compared to SWM |
| Contains interstitial neurons | Few or no interstitial neurons |

**Supplementary References**

1. Zanigni S, Evangelisti S, Testa C, et al. White matter and cortical changes in atypical parkinsonisms: A multimodal quantitative MR study [Internet]. Park. Relat. Disord. 2017;39:44–51.Available from: http://dx.doi.org/10.1016/j.parkreldis.2017.03.001

2. Chiang P-L, Chen H-L, Lu C-H, et al. White matter damage and systemic inflammation in Parkinson’s disease [Internet]. BMC Neurosci. 2017;18(1):48.[cited 2017 Nov 9 ] Available from: http://www.ncbi.nlm.nih.gov/pubmed/28595572

3. Du G, Lewis MM, Kanekar S, et al. Combined diffusion tensor imaging and apparent transverse relaxation rate differentiate Parkinson disease and atypical parkinsonism. Am. J. Neuroradiol. 2017;38(5):966–972.

4. Wang P-S, Yeh C-L, Lu C-F, et al. The involvement of supratentorial white matter in multiple system atrophy: a diffusion tensor imaging tractography study [Internet]. Acta Neurol. Belg. 2017;117(1):213–220.Available from: http://link.springer.com/10.1007/s13760-016-0724-0

5. Rulseh A, Keller J, Rusz J, et al. Diffusion tensor imaging in the characterization of multiple system atrophy [Internet]. Neuropsychiatr. Dis. Treat. 2016;Volume 12:2181–2187.[cited 2017 Sep 22 ] Available from: http://www.ncbi.nlm.nih.gov/pubmed/27616888

6. Yang H, Wang X, Liao W, et al. Application of diffusion tensor imaging in multiple system atrophy: the involvement of pontine transverse and longitudinal fibers [Internet]. Int. J. Neurosci. 2015;125(1):18–24.Available from: http://www.tandfonline.com/doi/full/10.3109/00207454.2014.896914

7. Worker A, Blain C, Jarosz J, et al. Diffusion tensor imaging of Parkinson’s disease, multiple system atrophy and progressive supranuclear palsy: A tract-based spatial statistics study. PLoS One 2014;9(11)

8. Prodoehl J, Li H, Planetta PJ, et al. Diffusion tensor imaging of Parkinson’s disease, atypical parkinsonism, and essential tremor [Internet]. Mov. Disord. 2013;28(13):1816–1822.[cited 2017 Sep 22 ] Available from: http://www.ncbi.nlm.nih.gov/pubmed/23674400

9. Surova Y, Szczepankiewicz F, Lätt J, et al. Assessment of Global and Regional Diffusion Changes along White Matter Tracts in Parkinsonian Disorders by MR Tractography. PLoS One 2013;8(6)

10. Tsukamoto K, Matsusue E, Kanasaki Y, et al. Significance of apparent diffusion coefficient measurement for the differential diagnosis of multiple system atrophy, progressive supranuclear palsy, and Parkinson’s disease: Evaluation by 3.0-T MR imaging. Neuroradiology 2012;54(9):947–955.

11. Focke NK, Helms G, Pantel PM, et al. Differentiation of typical and atypical Parkinson syndromes by quantitative MR imaging. Am. J. Neuroradiol. 2011;32(11):2087–2092.

12. Tha KK, Terae S, Yabe I, et al. Microstructural white matter abnormalities of multiple system atrophy: in vivo topographic illustration by using diffusion-tensor MR imaging. [Internet]. Radiology 2010;255(2):563–9.[cited 2017 Nov 8 ] Available from: http://pubs.rsna.org/doi/10.1148/radiol.10090988

13. Pellecchia MT, Barone P, Mollica C, et al. Diffusion-weighted imaging in multiple system atrophy: a comparison between clinical subtypes. [Internet]. Mov. Disord. 2009;24(5):689–96.Available from: http://www.ncbi.nlm.nih.gov/pubmed/19117367

14. Ito M, Watanabe H, Atsuta N, et al. Fractional anisotropy values detect pyramidal tract involvement in multiple system atrophy. J. Neurol. Sci. 2008;271(1–2):40–46.

15. Nicoletti G, Tonon C, Lodi R, et al. Apparent diffusion coefficient of the superior cerebellar peduncle differentiates progressive supranuclear palsy from Parkinson’s disease. Mov. Disord. 2008;23(16):2370–2376.

16. Seppi K, Schocke MFH, Prennschuetz-Schuetzenau K, et al. Topography of putaminal degeneration in multiple system atrophy: A diffusion magnetic resonance study. Mov. Disord. 2006;21(6):847–865.

17. Blain CRV, Barker GJ, Jarosz JM, et al. Measuring brain stem and cerebellar damage in parkinsonian syndromes using diffusion tensor MRI [Internet]. Neurology 2006;67(12):2199–2205.[cited 2017 Sep 22 ] Available from: http://www.ncbi.nlm.nih.gov/pubmed/17190944

18. Nicoletti G, Lodi R, Condino F, et al. Apparent diffusion coefficient measurements of the middle cerebellar peduncle differentiate the Parkinson variant of MSA from Parkinson’s disease and progressive supranuclear palsy. Brain 2006;129(10):2679–2687.

19. Shiga K, Yamada K, Yoshikawa K, et al. Local tissue anisotropy decreases in cerebellopetal fibers and pyramidal tract in multiple system atrophy. J. Neurol. 2005;252(5):589–596.
